# How to do quantitative myocardial perfusion cardiovascular magnetic resonance

**DOI:** 10.1093/ehjci/jeab193

**Published:** 2021-09-29

**Authors:** Noor Sharrack, Amedeo Chiribiri, Juerg Schwitter, Sven Plein

**Affiliations:** 1 Multidisciplinary Cardiovascular Research Centre and Biomedical Imaging Science Department, Leeds Institute of Cardiovascular and Metabolic Medicine, University of Leeds, Leeds LS2 9JT, UK; 2 School of Biomedical Engineering and Imaging Sciences, King’s College London, London SE1 7EH, UK; 3 Division of Cardiology, Cardiovascular Department, University Hospital Lausanne, Rue de Bugnon 46, CH-1011 Lausanne, Switzerland; 4 Cardiac MR Center, University Hospital Lausanne, Rue de Bugnon 46, CH-1011 Lausanne, Switzerland; 5 University of Lausanne, UniL, Faculty of Biology and Medicine, Rue du Bugnon 21, Lausanne, Switzerland

## Introduction

Myocardial perfusion cardiovascular magnetic resonance (CMR) using first-pass contrast-enhanced imaging is an established non-invasive test for the detection of myocardial ischaemia. Current practice involves visual interpretation of a series of dynamic images and relies on experienced reporters to identify perfusion defects. The acquired data can be used to derive quantitative maps of myocardial blood flow (MBF). Potential advantages over visual reading include removal of operator dependence, simpler and faster analysis, and the ability to detect disease with global rather than regional reduction of MBF. Recent developments allow semi-automated or fully automated in-line calculation of MBF. Although these methods remain mostly in the research domain, they are on the threshold of becoming integrated into routine clinical care. This ‘How to’ article gives a brief practical overview of the steps involved in generating quantitative MBF maps and suggests how these may be used in clinical practice. This article is not intended as an exhaustive review of the principles or clinical evidence, which have been summarised elsewhere.^1^

## General principles

Myocardial perfusion CMR acquires a dynamic series of images immediately after injection of a T1-shortening gadolinium-based contrast agent (GBCA). At least three myocardial short-axis slices are acquired, every 1–2 heartbeat(s) typically over 40–60 s using a T1-weighted dynamic pulse sequence. The in-plane spatial resolution should be at least 2.5 × 2.5 × 10 mm^3^, achieved by fast imaging techniques such as fast gradient echo imaging or steady-state free precession.

From these images, signal intensity profiles are taken from the left ventricular (LV) blood pool [to provide the arterial input function (AIF)] and the LV myocardium (which provides the tissue response). After conversion of dynamic MR signal changes to gadolinium contrast agent concentrations for the AIF and myocardium, quantitative perfusion (QP) assessment can be performed using a number of different models, providing MBF values in units of millilitres of blood per minute per gram of tissue. *Table 1* demonstrates steps to performing QP CMR.

**Table 1:** Steps to performing quantitative myocardial perfusion

Acquisition	Contrast bolus injection
	ECG triggered dynamic acquisition
	Proton density-weighted image
	Dual bolus or dual sequence
Signal processing	Respiratory motion correction
	Baseline signal correction
	Segmentation of arterial input function and tissue response
	Conversion of signal intensity profiles to Gd concentration profiles
	Modelling (quantification)
Output	MBF maps
	Rest MBF
	Stress MBF
	MBF reserve

ECG, electrocardiogram; Gd, gadolinium; MBF, myocardial blood flow.

## Contrast agent dose and delivery

For visual interpretation of myocardial perfusion CMR images, a dose of 0.05–0.1 mmol/kg GBCA is generally recommended to optimise signal changes in the myocardium. QP CMR additionally requires measurement of signal in the LV blood pool, where the concentration of GBCA is severalfold higher than in the myocardium and no longer linearly related to signal intensity. Uncorrected, this leads to miscalculation of MBF. To overcome this problem, in the ‘dual bolus’ method, a dilute bolus of contrast agent (typically 1/10 concentration of the full concentration bolus) is injected for LV blood pool analysis, followed by a full concentration bolus for myocardial analysis. In the ‘dual sequence’ method, images of the LV blood pool are acquired interleaved with the myocardial images following a single contrast bolus, but using a separate, less T1-sensitive pulse sequence, which avoids signal saturation. Once available outside of research settings, the dual sequence method is likely to integrate better into clinical workflow than the dual bolus method.

## Baseline corrections

In order to allow reliable modelling of QP and comparison between myocardial segments, raw signal intensity data needs to be corrected for baseline signal and surface coil inhomogeneities. This is typically achieved using proton density-weighted images or myocardial T1 mapping preceding the acquisition of perfusion data.

## Respiratory motion correction

Reliable QP analysis mandates elimination of bulk cardiac motion, usually achieved by breath-holding. However, long breath-holds can cause involuntary diaphragmatic drift and changes in heart rate. Furthermore, during pharmacological stress, patients may be unable to hold their breath reliably. These problems can be overcome by free-breathing acquisition methods with correction of bulk cardiac motion (rigid models) or more complex non-rigid deformation methods. As all motion correction methods can introduce artefacts, clinicians should always review both raw and motion-corrected images.

## Modelling

After the dynamic images are corrected for baseline signal and respiratory motion, signal intensity profiles are derived for the AIF and the myocardium using manual or automatic contouring. The AIF is typically taken from the basal LV but other sampling locations such as the ascending aorta have been proposed.

Endocardial and epicardial contours define the myocardium and further analysis can be on a global, segmental, or pixel basis. AIF and myocardial signal intensity profiles are converted to GBCA concentration time curves. Several mathematical models such as the Fermi function have been proposed for the final step of quantification of MBF, each with specific advantages and limitations that are beyond the scope of this article but have been reviewed elsewhere.^1^

## Interpretation and pitfalls of quantitative myocardial perfusion

The results of MBF quantification can be displayed at a segmental or pixel level with colour coding representing the magnitude of MBF. Rest and stress myocardial perfusion data are analysed separately to derive both rest and stress MBF. The ratio of stress/rest MBF defines the myocardial perfusion reserve (MPR), which can be displayed as a further polar plot. Further outputs from the analysis may include the AIF, myocardial signal intensity profiles, and other data that can be used for quality assurance.

A reduction in stress MBF or MPR implies either coronary artery disease (CAD) or coronary microvascular disease (CMD), but can also be caused by inadequate stress. Verification of adequate stress is typically achieved by reviewing the patient’s symptoms (flushing, breathlessness, and chest tightness), heart rate response (rise of ≥10 bpm), systolic blood pressure (fall of >10 mmHg) during the study, and splenic switch off on the acquired stress-images.^2^ However, this should not be used in isolation, and certain patient groups, particularly heart failure patients, may have a blunted haemodynamic response.

Dark rim artefacts affect the diagnostic accuracy of CMR perfusion and can mimic subendocardial perfusion defects leading to false-positive diagnosis of CAD. Using QP CMR, MBF is generally lower in true perfusion defects compared to dark rim artefacts, but may remain a source of error.

Registration, segmentation, and other errors may lead to erroneous MBF maps and automated and manual quality checks should be a routine part of quantitative myocardial perfusion imaging interpretation.

## Potential integration of QP into clinical reporting

No current guidelines exist on how to integrate quantitative myocardial perfusion CMR into clinical reporting and on how to combine it with visual analysis. As the bulk of the existing evidence for myocardial perfusion CMR is for visual analysis, this should continue to form the principal analysis strategy. QP maps may supplement visual interpretation in several ways:

1. Confirmation of visual read: Successive or simultaneous visual and quantitative analysis may enhance diagnostic certainty where the two strategies agree and, when results are discrepant, may alert the reader to the presence of artefacts.
2. Adequacy of haemodynamic response: When inadequate haemodynamic response is suspected based on a lack of clinical response or absence of splenic switch off, review of QP maps can help confirm (low-stress MBF and/or MPR) or refute (high-stress MBF and/or MPR) this suspicion.
3. Suspected CMD: Visual interpretation of myocardial perfusion CMR has limited ability to detect CMD. In patients with no regional visual perfusion defects and adequate haemodynamic response, but low-stress MBF or MPR on quantitative myocardial perfusion mapping, CMD is a likely diagnosis. Thresholds for diagnosing CMD with quantitative myocardial perfusion CMR have been proposed in small studies but have not been widely validated and may not be applicable across different acquisition methods.^3^
4. Disease extent: Visual read of myocardial perfusion CMR compares signal changes between different myocardial regions and is thus adjusted for the lowest perfused area in an image, potentially masking less severe defects elsewhere. Quantitative analysis provides objective absolute blood flow values for each region. This may be advantageous in multi-vessel CAD, where quantitative myocardial perfusion CMR may better identify disease extent than visual read.^4^
5. Follow-up studies and research: Quantitative myocardial perfusion CMR provides absolute numbers of MBF, which can help assess treatment effects.

**Figure 1:**
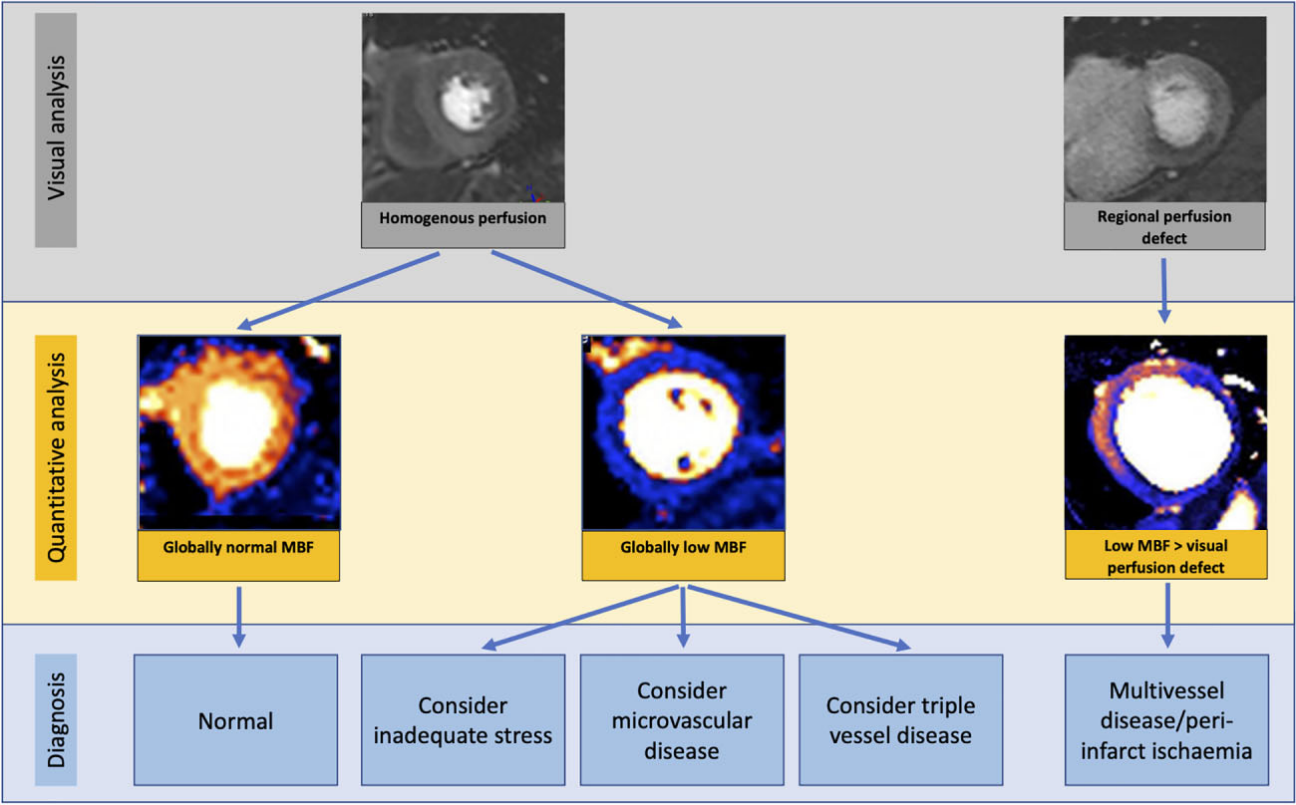
Potential scenarios when quantitative perfusion analysis may integrate into clinical practice. In a patient with visually homogenous perfusion, normal stress MBF/MPR by QP can reaffirm the diagnosis of a normal perfusion study. Conversely, visually homogenous perfusion with low-stress MBF/MPR suggests either inadequate vasodilatory response (check splenic switch off and haemodynamic response), coronary microvascular disease (which may show an endo to epicardial perfusion gradient), or severe triple vessel disease (where the pattern of perfusion is typically heterogeneous). In a patient with a regional perfusion defect on visual analysis, QP may help identify the extent of disease (visual analysis is relative and may underestimate disease extent) and peri-infarct ischaemia. MBF, myocardial blood flow.

## Automated in-line QP mapping

Historically, calculation of QP has been laborious, requiring manual contouring of hundreds of images, exporting data to external computers and manual analysis of the data with locally developed post-processing tools. This has prevented the use of quantitative CMR perfusion in routine clinical care. Recently, fully automatic in-line methods that include motion correction, automatic detection of the AIF, segmentation of the myocardium, and pixel-wise calculation of MBF have been proposed.^5^ Artificial intelligence can deliver automatic segmental and global quantification, allowing precise, rapid large-scale analysis. Such automated analysis pipelines can be integrated into the scanning acquisition to deliver QP maps in-line or immediately after data acquisition.

## Future directions

Large, multicentre prospective randomised-controlled studies are needed to further explore the prognostic value of quantitative myocardial perfusion CMR and its ability to guide revascularisation decisions. The most effective integration of QP into clinical pathways needs to be defined. Despite emerging consensus on the methodology, a number of different approaches to quantitative myocardial perfusion CMR are currently in use and international standardisation is needed.

**Conflict of interest:** J.Schwitter receives research support from Bayer Schweiz (AG). All other authors declared no conflict of interest.
